# Empirical validation of viral quasispecies assembly algorithms: state-of-the-art and challenges

**DOI:** 10.1038/srep02837

**Published:** 2013-10-03

**Authors:** Mattia C. F. Prosperi, Li Yin, David J. Nolan, Amanda D. Lowe, Maureen M. Goodenow, Marco Salemi

**Affiliations:** 1 University of Manchester, Faculty of Medical and Human Sciences, Northwest Institute of Bio-Health Informatics, Centre for Health Informatics, Institute of Population Health, Manchester, UK; 2 University of Florida, College of Medicine, Department of Pathology, Immunology and Laboratory Medicine, Gainesville, Florida, USA; 3 Florida Center for AIDS Research, Gainesville, Florida, USA; 4 Emerging Pathogens Institute, Gainesville, Florida, USA

## Abstract

Next generation sequencing (NGS) is superseding Sanger technology for analysing intra-host viral populations, in terms of genome length and resolution. We introduce two new empirical validation data sets and test the available viral population assembly software. Two intra-host viral population ‘quasispecies’ samples (type-1 human immunodeficiency and hepatitis C virus) were Sanger-sequenced, and plasmid clone mixtures at controlled proportions were shotgun-sequenced using Roche's 454 sequencing platform. The performance of different assemblers was compared in terms of phylogenetic clustering and recombination with the Sanger clones. Phylogenetic clustering showed that all assemblers captured a proportion of the most divergent lineages, but none were able to provide a high precision/recall tradeoff. Estimated variant frequencies mildly correlated with the original. Given the limitations of currently available algorithms identified by our empirical validation, the development and exploitation of additional data sets is needed, in order to establish an efficient framework for viral population reconstruction using NGS.

Next generation sequencing (NGS) is becoming an established experimental framework for genomics and clinical diagnostics^1^. NGS use covers a broad spectrum of applications, including *de novo* sequencing, re-sequencing, and metagenomics^2^. NGS has been employed successfully to characterize highly variable pathogens in different contexts^3^,^4^,^5^,^6^,^7^,^8^,^9^,^10^. The combination of huge coverage, deep resolution, primer- or shotgun-based sequencing, along with the possibility to analyze multiple samples in a single run, at a reasonable cost, makes NGS a valuable resource for diagnostic purposes^11^,^12^.

Several challenges have arisen concerning the raw NGS data processing, namely mapping to an appropriate reference sequence, error detection/correction^13^,^14^, single nucleotide variants (SNV) identification, and genome assembly methods^15^. In addition to sample processing and library design, problems for computational assembly and SNV detection of long haploid/diploid genomes (several thousands of kilo-bases and above) include genome coverage, repeated regions, and genome rearrangements. Distinct complexities arise with viral populations or so-called ‘quasispecies’^16^,^17^. Quasispecies are composed of different proportions of distinct variants, some at very low frequency, that are often subject to recombination. One supposes here that the experimental procedures, including polymerase chain reaction amplification, minimize the chance to generate *in vitro* recombinants. When processing a quasispecies with NGS, it is vital to correctly recognize minority SNV which can be close to the sequencing error rate. Another challenge is to correctly assemble whole-gene or whole-genome variants, along with their proportions, without generating spurious *in silico* recombinants.

The current approach for analyzing a whole-gene or whole-genome quasispecies relies on clonal Sanger sequencing. While clonal sequencing has the advantage of an almost perfect variant reconstruction, given an appropriate primer design, the approach is not readily automatable, and cumbersome in terms of bench-work. In principle, NGS will overcome Sanger's limitations. A key step will be when available SNV detection and assembly algorithms are sufficiently reliable. Approaches for SNV analysis that can identify minority variants have been devised^8^,^18^,^19^,^20^. Several methodologies for quasispecies assembly, all based on reference mapping and overlap-graph paradigm, have been also introduced^21^,^22^,^23^,^24^,^25^,^26^,^27^,^28^,^29^,^30^, along with software implementations, tested both on simulated and quasi-empirical data, such as pooled inter-host samples^18^ or artificially created mixtures^19^. Performance of quasispecies reconstruction algorithms depend on many biological factors: the efficacy of error correction methods, overlap lengths, assembly heuristics, including the quasispecies diversity and of the extent of heterogeneity. It is of vital importance to assess the efficacy of quasispecies assembly methods with real data sets in order to test their use for diagnostics in a clinical context.

This work introduces two new empirical validation data sets and tests quasispecies assembly algorithms. From the plasma samples of two patients infected with type-1 human immunodeficiency or hepatitis C viruses (HIV-1, HCV), using Sanger sequencing, plasmids isolated from transformed bacterial cultures were mixed at controlled proportions, and then the mixtures were shotgun-sequenced with the Roche's 454 sequencing platform. Performance of four different assembly algorithms, including QuRe, PredictHaplo, Geneious™ *de novo*, and ShoRAH (see Beerenwinkel *et al.*^26^ for a comprehensive review) were evaluated in terms of phylogenetic clustering with the original Sanger clones, precision/recall indices, diversity and recombination analysis.

## Results

The HCV data set comprised 16 sequences encompassing 669 bases of envelope regions E1–E2. The average diversity was 6.47%. Variant prevalence was approximately [41%, 28%, 13%, 5%, 3%, 3%, 2%, 2%, 1%, 1%, 0.2%, 0.2%, 0.1%, 0.1%, 0.1%, 0.1%]. The optimal nucleotide substitution model, according to the Akaike information criterion (AIC), was the Tamura 3-parameter (1992), with a γ-distributed rate of heterogeneity parameterized on α = 0.28. The HIV-1 data set comprised of 20 *env* sequences (1,409 bases of the gp120 regions V1–V5), with an average diversity of 4.7%. Variant prevalence was approximately [18%, 13%, 13%, 10%, 10%, 8%, 8%, 5%, 5%, 3%, 3%, 1%, 1%, 0.6%, 0.6%, 0.3%, 0.3%, 0.1%, 0.1%, 0.1%]. The optimal nucleotide substitution model according to AIC was the Tamura-Nei (1993), with a γ-distributed rate of heterogeneity parameterized on α = 0.32.

The Roche's 454 platform (GS FLX Titanium XLR70) yielded 156,655 reads for the HCV and 153,670 for the HIV-1 plasmid mixture, sequenced on two separate lanes. The average (st.dev.) read length was 363 (109) for HCV and 346 (98) for HIV. Using QuRe, 27,912 (17.82%) and 53,930 (35.09%) reads were successfully mapped (and trimmed) to the HCV and HIV-1 reference sequences, respectively. The length ratios between the sequenced regions and the plasmid were ~ 16% for HCV and ~ 39% for HIV-1. The average (st.dev.) coverage of each mapped base was 9,553 (1,881) for HCV and 9,565 (4,216) for HIV. All the reads were also mapped to the plasmid reference sequence and a random subset of 70,000 reads was retained from both experiments to assess sequencing error rates.

### Error rate assessment and correction

Out of the two 70,000 random read sets selected from both experiments, 49,866 (71.24%) of the HCV and 62,303 (89.00%) of the HIV-1 mixture were retained when mapped (and trimmed) to the plasmid reference. Note, the observed differences in plasmid mapping and sequence recovery are because two different insertion points were used, whilst the same linear plasmid reference sequence was used for both mappings.

The overall mean error prevalence (standard error, SE) using reads from the HCV mixture was 1.26% (0.010). Insertions, deletions and mismatches accounted for 0.40% (0.005), 0.39% (0.005), and 0.47% (0.006) of the total, respectively. As expected errors were more frequent in homopolymeric regions: 1.33% (0.017) and 1.22% (0.012), respectively. When mapping reads from the HIV-1 mixture, overall error prevalence (SE) was 1.30% (0.006). Base insertions, deletions and mismatches accounted for 0.36% (0.003), 0.47% (0.004), and 0.47% (0.004) of the total, respectively. Error prevalence (SE) in homopolymeric regions was 2.14% (0.016) and 1.05% (0.006) in non-homopolymeric regions.

Using the error correction method embedded in QuRe, the overall error was decreased by 85.29% (6.80 fold) in the HCV sample and by 69.77% (3.54 fold) in the HIV-1 sample. With ShoRAH, the decrease was 85.96% (7.12 fold) for HCV and 83.95% (6.66 fold) for HIV-1. Table 1 reports error rates for the two data sets and correction methods, overall and stratified by error type (insertions, deletions, mismatches and homopolymeric regions).

### Quasispecies assembly and evaluation of reconstruction performance

QuRe, PredictHaplo, ShoRAH and Geneious™ *de novo* were applied to both NGS data sets, using the corresponding HIV-1/HCV reference sequences. QuRe used the homopolymeric and non-homopolymeric error rates as estimated from mapping the reads to the plasmid reference (default parameters for the rest). The other programs were run with default parameters.

**Figure 1:**
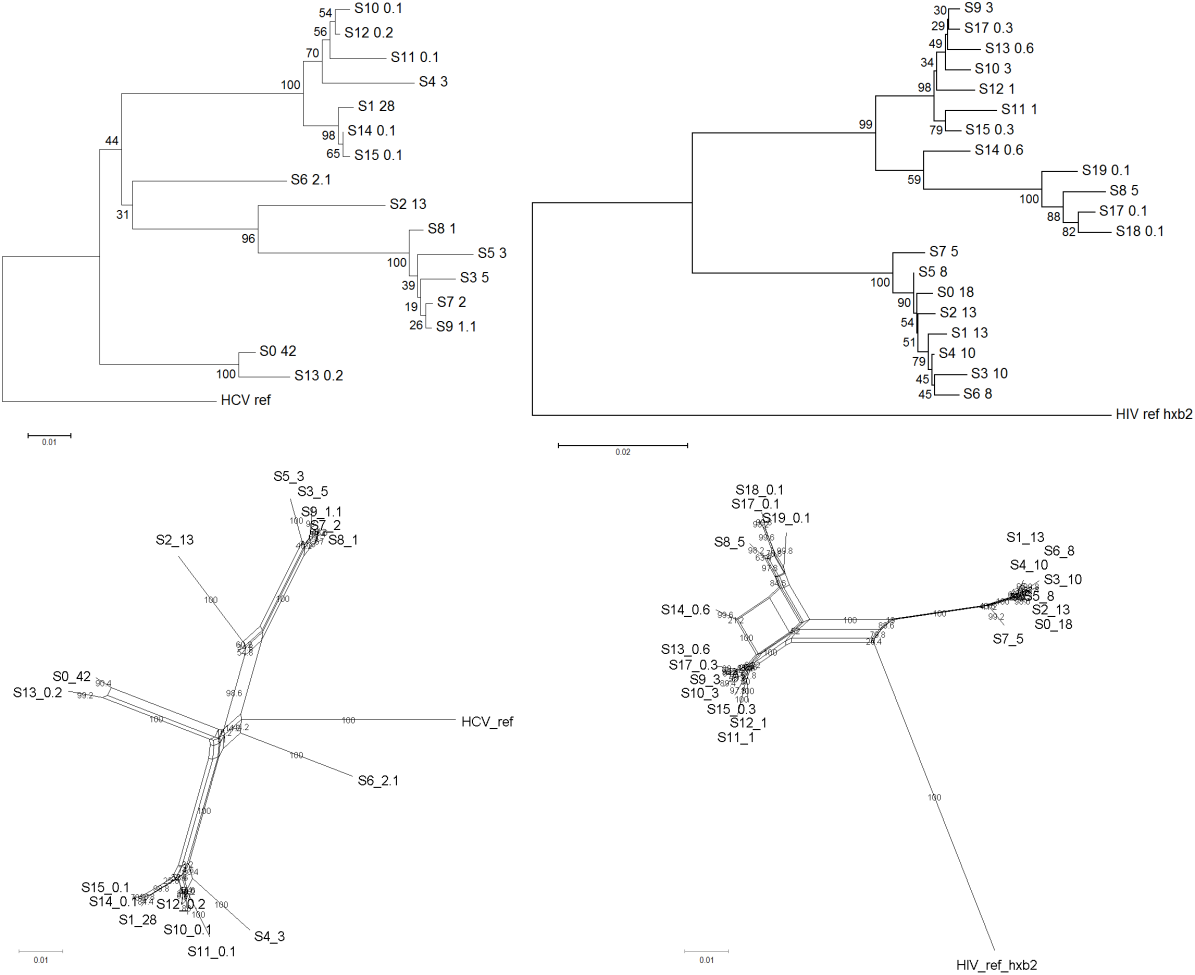
Phylogenetic trees (upper panels) and networks (lower panels) of the original Sanger sequences for the HCV (left panels) and HIV-1 (right panels) data sets. Neighbor-joining and neighbor-net algorithms were run on optimized models of evolution, over 500 bootstrap runs. Node labels show bootstrap percentages. Numbers after the labels represent variant prevalence (%).

QuRe, PredictHaplo, ShoRAH and Geneious™ *de novo* yielded 9, 4, 200, and 81 distinct variants respectively for the HCV data set, and 9, 4, 1,247, and 159 variants for HIV-1 data set. Tables 2 and 3 report precision/recall performance of each reconstruction algorithm, population characteristics and recombination tests. QuRe and PredictHaplo exhibited the highest precision (between 0.55 and 1.00), whilst ShoRAH had the highest recall (0.3 to 0.5). Reconstructed variants were classified as ‘correct’ when clustering with original Sanger clones in a phylogenetic tree at ≥ 75% bootstrap support (see Methods). Of note, when inferring a phylogeny from the sole Sanger data set, the proportion of highly-supported nodes (>75% bootstrap) was 43% and 50%, for HCV and HIV-1, respectively (Figure 1, upper panels). Therefore placement for some variants in the tree was ambiguous (i.e. some variants were probably too similar to distinguish).

Detection of recombination, using the pairwise homoplasy index test (PHI-test), was marginally significant in the HCV Sanger data set (*P* = 0.043), and highly significant in the HIV-1 data set (*P* < 0.0001). Recombination networks are shown in Figure 1 (lower panels). QuRe and PredictHaplo yielded the lowest number of *in silico* recombinants, showing a moderate decrease of the PHI-test *P*-value. In terms of variant frequency estimation, all methods correlated significantly with the original distributions, with Pearson's linear correlation ranging from 0.3 to 0.6 (*P* < 0.0001).

**Figure 2:**
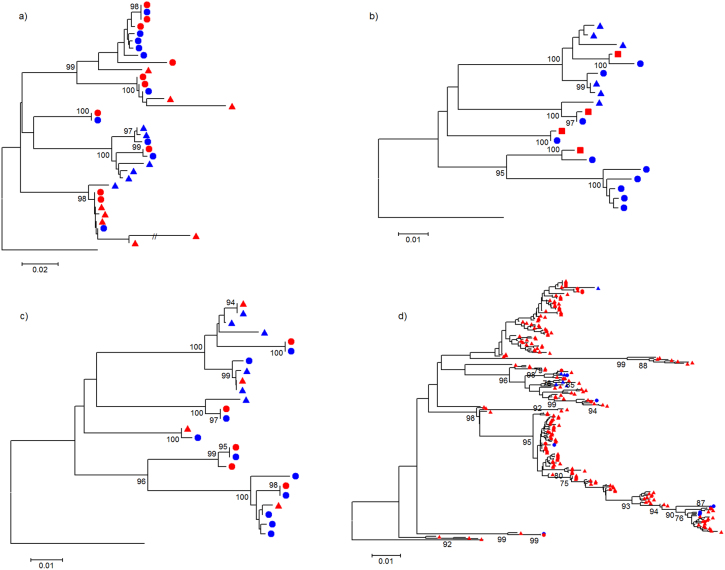
Evolutionary history inferred by neighbor-joining, using an optimized nucleotide substitution model, that compares HCV variants reconstructed by each quasispecies assembler with the original Sanger clones; trees are rooted using the mapping reference sequence. Panels (a), (b), (c), and (d) show Geneious™ *de novo*, PredictHaplo, QuRe and ShoRAH, respectively. Node numbers represent% bootstrap replicates (of 500) ≥ 75%. Bullets represent variants at a frequency ≥ 5%, and triangles those < 5% (not available for PredictHaplo, shown by squares). Blue color indicates Sanger isolates, and red reconstructed variants.

**Figure 3:**
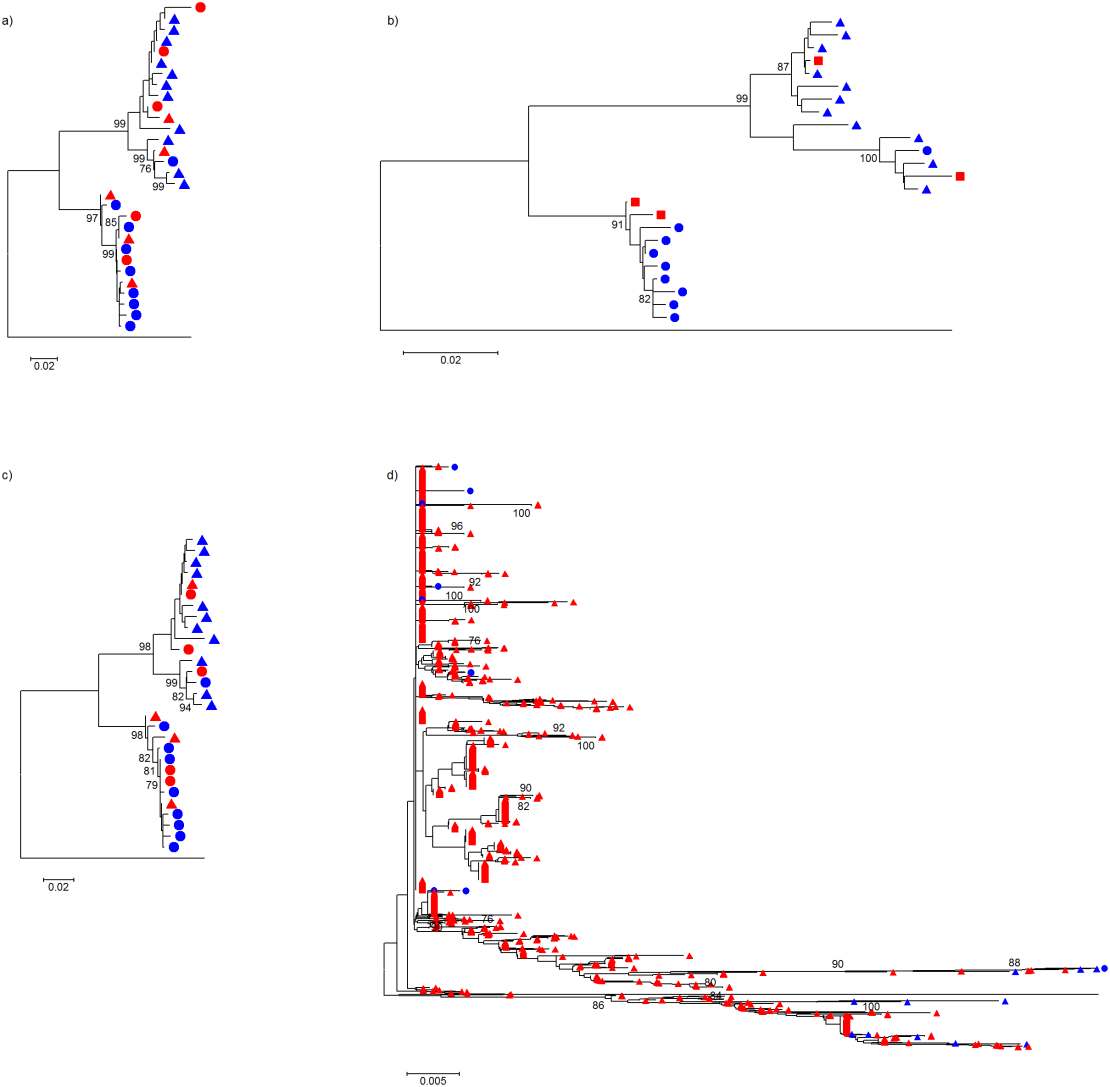
Evolutionary history inferred by neighbor-joining, using an optimized nucleotide substitution model, that compares HIV-1 variants reconstructed by each quasispecies assembler with the original Sanger clones; trees are rooted using the mapping reference sequence. Panels (a), (b), (c), and (d) show Geneious™ *de novo*, PredictHaplo, QuRe and ShoRAH, respectively. Node numbers represent% bootstrap replicates (of 500) ≥ 75%. Bullets represent variants at a frequency ≥ 5%, and triangles those < 5% (not available for PredictHaplo, shown by squares). Blue color indicates Sanger isolates, and red reconstructed variants.

**Figure 4:**
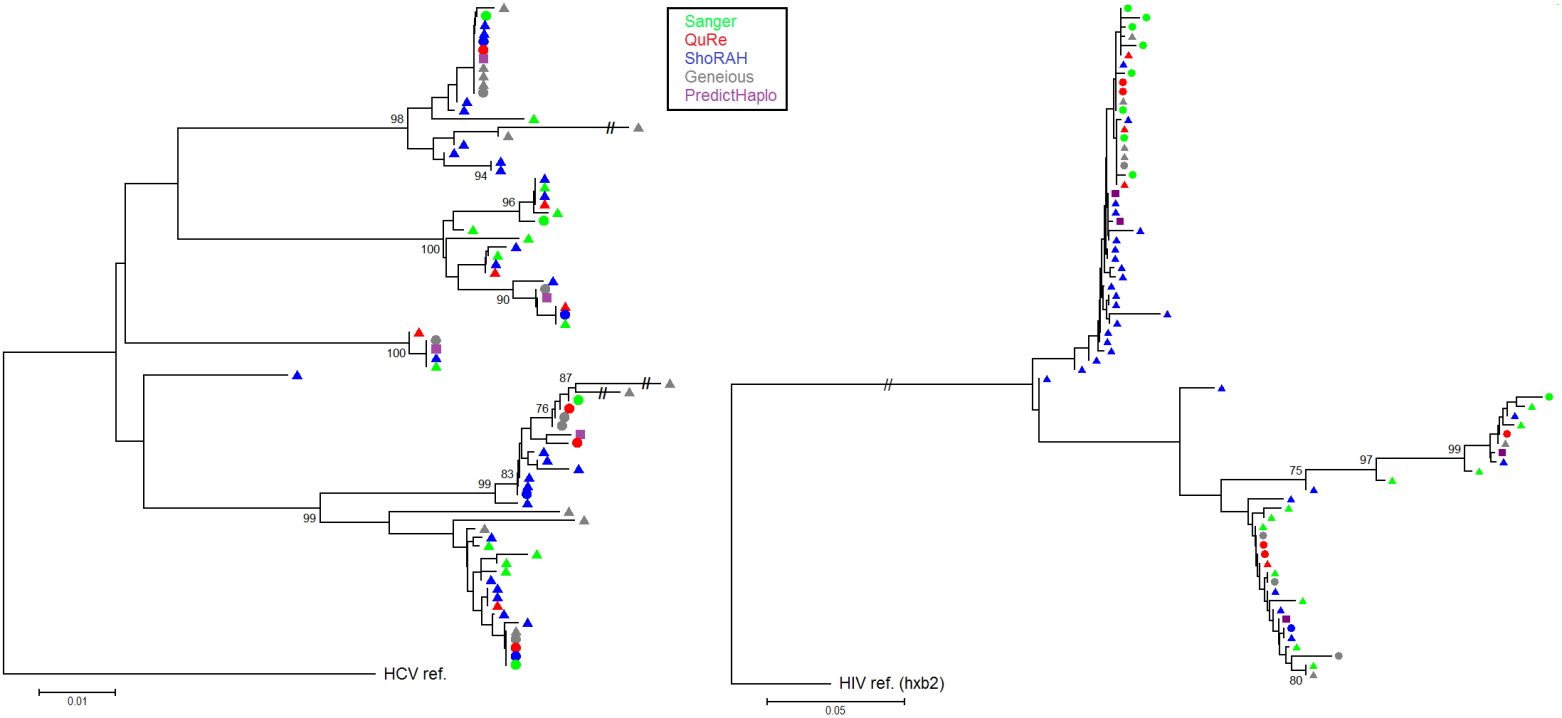
Phylogenetic trees comparing together all reconstructed variants from different assemblers with the original Sanger sequences for the HCV and HIV-1 experiments (left and right panels, respectively). Trees have been inferred using neighbor-joining on an optimized model of evolution, rooted on the mapping reference sequence, performing 500 bootstraps (nodes with ≥ 75% bootstrap support are shown). For ease of read, only the 30 highest-frequency variants from ShoRAH have been included.

Figure 2 depicts phylogenetic trees for the HCV experiment that compare variants output by each assembler together with the original Sanger sequences. Trees for the HIV-1 experiment are shown in Figure 3. Figure 4 shows trees for the two data sets (left and right panels for HCV and HIV-1, respectively) inferred by combining all variants from all algorithms and original Sanger sequences. Although reconstruction performance was not optimal in most cases, the same –overall- population structure can be found across all trees.

## Discussion

This work evaluated the performance of viral quasispecies assembly algorithms in an empirical setting. Comparisons were performed using both an HCV and HIV-1 data sets, sequencing plasmid clones with Sanger technology and mixing them at known proportions. Plasmid mixtures were shotgun-sequenced using Roche's 454 platform.

The estimated error rates were similar in the HIV-1 and HCV samples. The overall error prevalence was consistent with previous estimates^10^,^13^,^14^, as well as the higher prevalence of errors in homopolymeric regions, and the higher prevalence of insertions and deletions as compared to mismatches (although the ratio varied across previous studies). ShoRAH provided a better error reduction as compared to QuRe. Note that the error correction was evaluated only in terms of removing mismatches or indels from the plasmid reference sequence, and not on the ability to distinguish true signal from false signal, especially in presence of minority changes. As a consequence, error correction should be evaluated also on data sets where true mutations exist and assess precision/recall rates at different frequency thresholds. Additionally, the read filtering, mapping, and error counting was carried out with QuRe, which potentially gives QuRe an advantage in the error correction phase. This is because QuRe performed error correction after mapping, while ShoRAH corrected reads before the mapping. Then ShoRAH reads were re-mapped with QuRe (not enabling its error correction module) in order to obtain comparable output.

All of the quasispecies assembly methods published to-date have performed robustly in simulations and empirical experiments^18^,^23^,^31^,^32^. This study is the first to consider actual intra-patient quasispecies, sequenced with Sanger, mixed at controlled proportions and then processed using NGS. Previously, validation on real data had been performed using inter-patient samples^18^,^23^. A recent work also used a controlled proportion approach to assess performance of error correction algorithms, but an artificial quasispecies was generated^19^. Here, both for HCV and HIV-1 data sets, all assemblers were able to capture a proportion of the most divergent lineages as indicated by the phylogenetic analysis. PredictHaplo was the most conservative algorithm, with the least number of variants reported, while ShoRAH yielded the highest number. QuRe and PredictHaplo exhibited the highest precision, whilst ShoRAH had the highest recall. QuRe showed the best precision/recall tradeoff. Estimated variant frequencies mildly correlated with the original values. *In silico* recombination was more pronounced in the output by ShoRAH and Geneious™ *de novo*. Geneious™ *de novo* was the least effective assembler in terms of precision and recall, but when considering only long contiguous sequences its performance increased substantially. Note that originally Geneious™ *de novo* has not been designed to assemble a quasispecies. In regards to the parameter set-up of assemblers, PredictHaplo did not require complex tuning and results were similar across different runs. ShoRAH may have benefited from a parameter optimisation (especially the sliding window sizes, but also number of iterations and the hyper-parameter α), but the search space would have required a considerable computational burden. QuRe required only a few parameters (error rates, estimated from the plasmid data, and number of iterations) and the rest was auto-optimised during the process. For all variants reconstructed by the algorithms, the cutoff for clustering with a Sanger clone was set to 75%, without a fixed threshold on nucleotide differences. Results may change when the cutoff or distance criteria are altered.

To determine the performance of reconstruction algorithms, one has to evaluate their output in different contexts. Performance of programs can be also subject to error patterns proper of NGS machinery. Besides the experimental yield, i.e. read length and coverage, factors to consider include: the number of variants in the population, the variant prevalence, the average diversity, the rate of heterogeneity, and the recombination signal. We look forward to testing methodological approaches for quasispecies assembly that specifically incorporate recombination, such as the recent QuasiRecomb, which currently performs only local reconstruction^33^.

Evaluation of quasispecies assemblers in different scenarios, using both simulated and empirical data, will be an important step in characterizing the clinical relevance of viral populations in infected individuals.

## Methods

### Sample preparation and processing

Plasmids containing HIV-1 *env* gp120 V1–V5, or genotype 1 HCV envelope E1–E2 sequences, derived from virus in the plasma of infected patients, previously published by Gray et *al.*^34^ and Ho *et al.*^35^, isolated from transformed bacterial cultures, were mixed at controlled decreasing proportions (from 42% to 0.1%, see the Results section). Sample preparation and processing procedures were based on the previous work of Zagordi *et al.*^18^. The cloning kit used was Invitrogen TOPO® TA Cloning® Kit (with pCR®2.1-TOPO® Vector) with One Shot® Mach1™ T1 Phage-Resistant Chemically Competent *E. coli* (http://products.invitrogen.com/ivgn/product/K451020).

Study design and sample usage for viral genotyping was approved by University of Florida Institutional Review Board. NGS was performed at University of Florida using the Roche's GS FLX Titanium XLR70 platform. Raw NGS data are available for free upon formal request to the authors.

### Mapping to a reference and quasispecies assembly

The HCV reference used for read mapping was the H77 (Genbank NC_004102.1), encompassing 669 bases of the two envelope regions E1–E2 (positions 1,315- 1,984 relative to the full-genome numbering). The HIV-1 reference sequence was the HXB2 (Genbank K03455) *env* gp160 (positions 6,225–8,792 relative to the full-genome numbering).

Reads were mapped to each reference using QuRe^32^, a multi-threaded, platform-independent software designed for read mapping, error correction and quasispecies assembly. QuRe uses the JAligner (http://jaligner.sourceforge.net/) implementation of the Smith-Waterman-Gotoh local alignment algorithm^36^ for mapping reads and discards those that do not exhibit an alignment score sufficiently high as compared to a quasi-random score distribution, according to a *z*-test as discussed by Bacro and Comet^37^. For this study, gap open and extension penalties of 23 and 0.3 were chosen, respectively. Reads that spanned both a part of the viral insert and the plasmid were trimmed by the local alignment and retained if the statistical test was below the chosen threshold (*P* < 0.01 by default, corrected with the Benjamini-Hochberg procedure). Therefore, a read could be mapped to both the plasmid reference and the viral reference.

Error correction was performed using QuRe^32^ and ShoRAH^30^. The former implemented the method by Wang *et al.*^8^, which assumed a Poisson distribution of errors parameterized differently in homopolymeric and non-homopolymeric regions. A position in the reference sequence was defined as homopolymeric if the substring generated by elongating three bases to the left and three bases to the right of that position contained at least three consecutive bases of the same type. ShoRAH implemented a Bayesian probabilistic clustering over sliding windows across the reference sequence, correcting reads by assigning each one to the closest cluster^25^. Errors were calculated overall and stratified by type (insertion, deletions, mismatches, and homopolymeric vs. non-homopolymeric region).

Quasispecies assembly was executed employing QuRe v0.9994 (http://sourceforge.net/projects/qure/), ShoRAH v0.5.1 (http://www.bsse.ethz.ch/cbg/software/shorah), PredictHaplo v0.4 (http://bmda.cs.unibas.ch/HivHaploTyper/), and the Geneious™ *de novo* assembler (http://www.geneious.com/). The quasispecies reconstruction method of QuRe employs a method based on an overlap graph constructed over sliding windows, selecting candidate variants using an algorithm based on overlap consistency and similarity of frequency distributions of variants in each window^23^,^32^. ShoRAH perform a parsimony-based reconstruction after the sliding window Bayesian clustering^30^, whilst PredictHaplo extends the sliding window Bayesian clustering approach to a global quasispecies inference based on a hidden Markov model.

### Phylogenetic, recombination analysis and performance assessment

Reconstructed variants were aligned with the original Sanger sequences and a phylogenetic/recombination analysis was carried out using the MEGA software v5.05^38^ and SplitsTree v4^39^, as follows. In MEGA, a multiple alignment was obtained using MUSCLE^40^, then an optimal nucleotide substitution model was selected by minimizing the AIC, and finally bootstrapped (n = 500) neighbor-joining trees were inferred. The multiple alignment was used also as input for the recombination analysis in SplitsTree, using the PHI-test by Bruen *et al.*^41^, and generating phylogenetic networks with the bootstrapped (n = 500) neighbor-net algorithm.

By looking at the inferred phylogenetic trees, an assembled variant that was clustering with an original Sanger clone with ≥ 75% of bootstrap support was considered as a correct reconstruction. If more than one reconstructed variant was clustering with one or more original Sanger clones, this was counted as a unique correct reconstruction. A minimal nucleotide difference threshold was not fixed. This criterion was chosen to account for the fact that assemblers may output variants of different lengths and a subsequent distances obtained from the multiple alignments may be biased towards longer/shorter sequences. Precision (# of correctly reconstructed variants/# total number of reconstructed variants) and recall (# of correctly reconstructed variants/# number of original variants) were used as performance indicators, as well as the *P*-value of recombination as obtained by the PHI-test.

## Author Contributions

M.C.F.P. conceived the study, executed computational analyses and wrote the manuscript; L.Y. designed the laboratory experiments; D.J.N. and A.D.L. performed experiments; M.M.G. supervised the experiments and reviewed the manuscript; M.S. executed phylogenetic analysis and reviewed the manuscript.

## Figures and Tables

**Table 1:** Raw and corrected (QuRE, ShoRAH) estimated error rates for the Roche's GS FLX Titanium XLR70, as estimated by shotgun-sequencing the plasmid TOPO2.1 with HCV/HIV-1 inserts, and mapping the reads against the TOPO2.1 reference using the QuRe read aligner/filter module

				error prevalence (standard error) by type				
data set (n = 70,000)	mapped (n;%)	error correction method	overall error prevalence (standard error)	insertions	deletions	mismatches	homopolymeric	non-homopolymeric
HCV	49,866; 71.24%	non-corrected	1.26% (0.0096)	0.40% (0.0054)	0.39% (0.0054)	0.47% (0.0059)	1.33% (0.0168)	1.22% (0.0117)
		QuRe	0.19% (0.0037)	0.02% (0.0012)	0.14% (0.0032)	0.02% (0.0014)	0.27% (0.0076)	0.14% (0.0040)
		ShoRAH	0.18% (0.0037)	0.00% (0.0000)	0.12% (0.0031)	0.06% (0.0021)	0.24% (0.0073)	0.14% (0.0041)
HIV-1	62,303; 89.00%	non-corrected	1.30% (0.0060)	0.36% (0.0032)	0.47% (0.0037)	0.47% (0.0036)	2.14% (0.0162)	1.05% (0.0062)
		QuRe	0.39% (0.0033)	0.06% (0.0013)	0.25% (0.0027)	0.08% (0.0015)	0.95% (0.0108)	0.23% (0.0029)
		ShoRAH	0.21% (0.0025)	0.00% (0.0001)	0.13% (0.0020)	0.07% (0.0015)	0.47% (0.0079)	0.13% (0.0022)

**Table 2:** Performance of quasispecies assembly algorithms

data set	Assembly method	# variants output	# correct variants	Precision	Recall
HCV (n = 16)	ShoRAH	200	8	0.04	0.50
	QuRE	9	8	0.89	0.50
	PredictHaplo	4	4	1.00	0.25
	Geneious™ *de novo*	81 (18)*	5	0.06 (0.28)*	0.31
HIV-1 (n = 20)	ShoRAH	1,247	6	0.005	0.3
	QuRE	9	5	0.55	0.25
	PredictHaplo	4	3	0.75	0.15
	Geneious™ *de novo*	159 (10)*	4	0.02 (0.40)*	0.20

*considering only contigs > 500 (for HCV) and > 900 (for HIV-1) base pair long.

**Table 3:** Population characteristics of the original Sanger and of the assembled quasispecies (post-alignment figures after gap-stripping)

Data set	method	# variants output	PHI-test for recombination (*P*-value)	Average diversity (p-distance)	Minimal diversity (p-distance)	γ-distributed rate of heterogeneity (α)	effective population size	Segregating sites	Parsimony info
HCV	original Sanger clones	16	*P* = 0.043	0.0647	0.001	0.47	0.83	19%	12%
	ShoRAH	200	*P* = 0.0002	0.0531	0.000	0.22	0.16	28%	26%
	QuRE	9	*P* = 0.023	0.0689	0.007	0.19	0.21	17%	11%
	PredictHaplo	4	*P* = 0.03	0.0748	0.068	0.05	0.56	13%	3%
	Geneious™ *de novo*	81 (18)*	*P* < 0.0001	0.078	0.000	0.52	0.31	36%	12%
HIV-1	original Sanger clones	20	*P* < 0.0001	0.047	0.002	0.09	13.26	14%	10%
	ShoRAH	1,247	*P* < 0.0001	0.0109	0.000	0.17**	116.77	36%	29%
	QuRE	9	*P* < 0.0001	0.0425	0.000	0.10	44.72	10%	6%
	PredictHaplo	4	*P* < 0.0001	0.0685	0.016	0.05	20.09	12%	4%
	Geneious™ *de novo*	159 (10)*	*P* < 0.0001	0.0524	0.000	0.57	41.64	19%	8%

*considering only contigs > 500 (for HCV) and > 900 (for HIV-1) base pair long.

**estimated on a random subset of 200 sequences.
